# Salvage of necrotic flap following malignant peripheral nerve sheath tumor excision using multimodal pharmacotherapy: a case report

**DOI:** 10.3389/fonc.2025.1689834

**Published:** 2025-10-15

**Authors:** Lili Chen, Xiangyi Wu, Feng Li

**Affiliations:** ^1^ Longhua Hospital affiliated to Shanghai University of Traditional Chinese Medicine, Shanghai, China; ^2^ Department of Plastic and Reconstructive Surgery, Shanghai Ninth People’s Hospital, Shanghai Jiao Tong University School of Medicine, Shanghai, China

**Keywords:** malignant peripheral nerve sheath tumor, misdiagnosis, flap necrosis, wound repair, Chinese medicine

## Abstract

A 73-year-old man presented with a 20-year history of a mass in the right scapular region that had rapidly enlarged and become painful. Initially misdiagnosed as an infected sebaceous cyst, intraoperative findings revealed a solid, poorly demarcated mass. Histopathology confirmed malignant transformation of a neurofibroma into a high-grade malignant peripheral nerve sheath tumor, with positive margins. The patient underwent wide local excision with frozen-section–verified clear margins and full-thickness back flap reconstruction. On postoperative day 3, the flap developed progressive ischemic necrosis. Salvage was achieved using a novel combination of topical papaverine hydrochloride, Danhong injection, and enzymatic debridement, leading to full revascularization by day 9. The patient was discharged in stable condition on day 15. This case underscores the need for early pathological evaluation of rapidly enlarging skin masses when clinical and imaging findings are discordant. It also demonstrates the successful use of papaverine, Danhong, and enzymatic debridement as a salvage strategy for postoperative flap necrosis.

## 1 Introduction

MPNST is a rare and aggressive soft tissue sarcoma that accounts for 5% to 10% of soft tissue malignancies and is commonly associated with patients with NF1 or prior radiotherapy exposure (1). The aggressive nature confers a 5-year survival rate of 30%-50%, with high risks of local recurrence and distant metastasis (2–4).

## 2 Case description

A 73-year-old male presented with a 20-year history of a slowly enlarging right scapular region mass. Initially diagnosed as a sebaceous cyst and managed conservatively, the lesion acutely increased in size with associated pain and erythema over one week. Physical examination revealed a 6x6 cm firm, erythematous, tender mass near the right posterior axillary line at the ninth thoracic vertebra level. Color Doppler ultrasound identified an ill-defined, heterogeneous hypoechoic subcutaneous nodule (61x26 mm) with peripheral vascularity, suggestive of an infected sebaceous cyst.

Under local anesthesia, incision and drainage were attempted. Intraoperatively, no purulent material was encountered; instead, a solid subcutaneous mass was identified.

**Figure 1:**
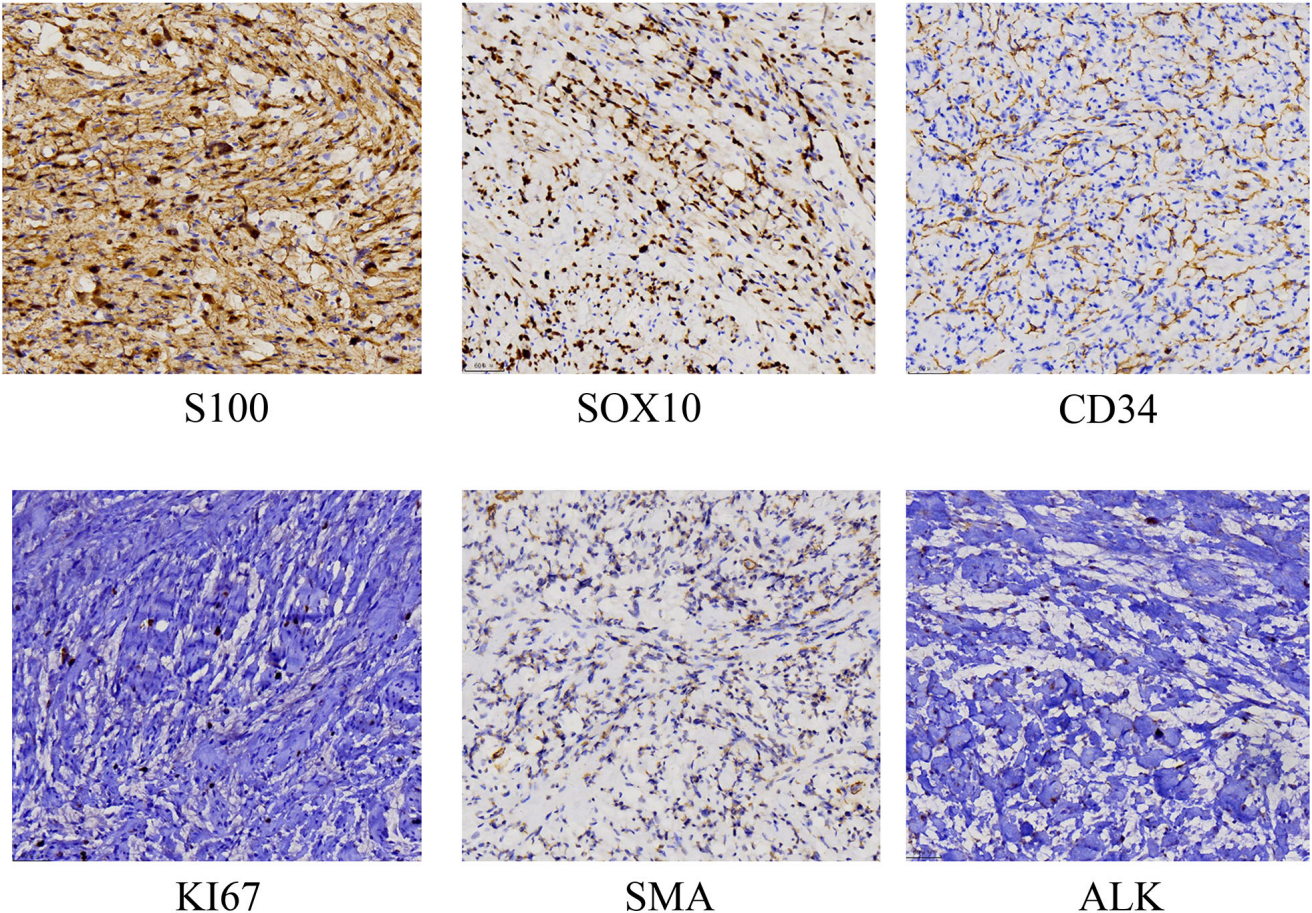
The immunohistochemical findings of the postoperative pathological tissue.

**Figure 2:**
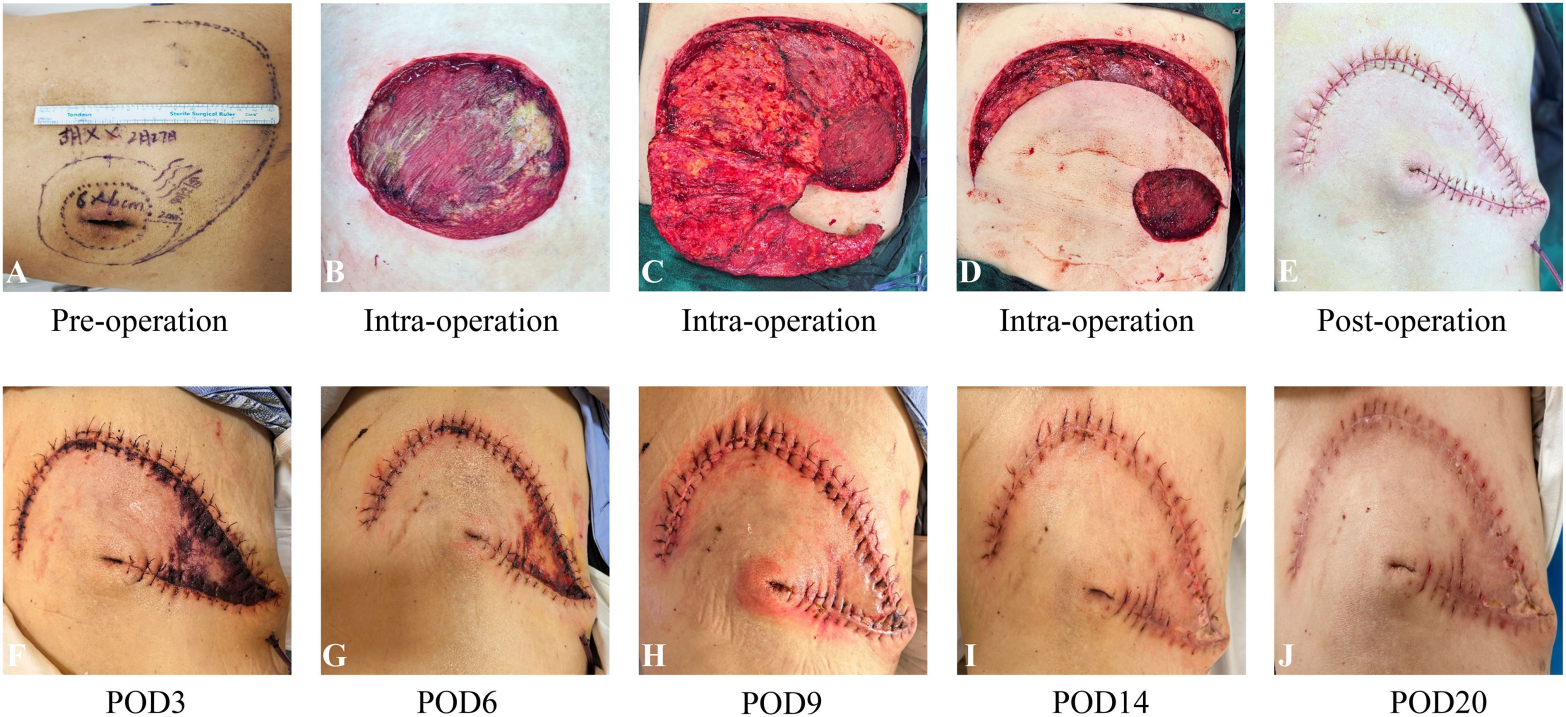
Skin manifestations, clinical course and timeline flap necrosis and subsequent recovery. **(A)** preoperative photograph. **(B–D)** intraoperative photographs showing flap design. **(E)** postoperative photograph after wound closure. **(F)** postoperative day 3, distal flap discoloration noted, indicating early signs of ischemia. **(G–J)** process of flap salvage.

Histopathology revealed malignant transformation of a neurofibroma into a high-grade malignant peripheral nerve sheath tumor (MPNST) with involvement of lateral and deep margins. Immunohistochemistry supported the diagnosis (S-100 positive, Ki-67 10%, SMA negative, CD34 positive, ALK positive) (**Figure 1**). Additional imaging studies excluded distant metastasis and provided valuable information for determining the extent of surgery. Wide local excision was subsequently performed. A 2-cm margin was excised down to the latissimus dorsi, and tumor-free margins were confirmed from four quadrants by intraoperative frozen sections. Due to the large defect, a rotating flap is designed under ultrasound positioning during the operation (**Figures 2A–E**).

On postoperative day 3, flap necrosis involving approximately 30% of the area was observed, which exhibited signs of progressive ischemic compromise, characterized by darkening of the skin, diminished capillary refill, and advancing necrosis. Salvage therapy was initiated immediately: intravenous use of papaverine hydrochloride injection (30mg tid) and Danhong injection (20ml qd) was started. At the same time, the dressing regimen was adjusted to cover the flap with a biological enzyme gauze dressing (ShaBuChuangMianFuLiao, 7.5cm×7.5cm (four layers)). On postoperative day 6, perfusion improved and 50% flap revascularization was achieved. On the 9th day, the flap demonstrated complete revascularization with restoration of normal color and turgor. On the 14th day after surgery, intermittent suture removal was started (**Figures 2F–J**). For 30 days after surgery, the skin healed completely. The patient was discharged in stable condition. The diagnosis and treatment timeline is shown in **Table 1**.

**Table 1:** Diagnosis and treatment timeline.

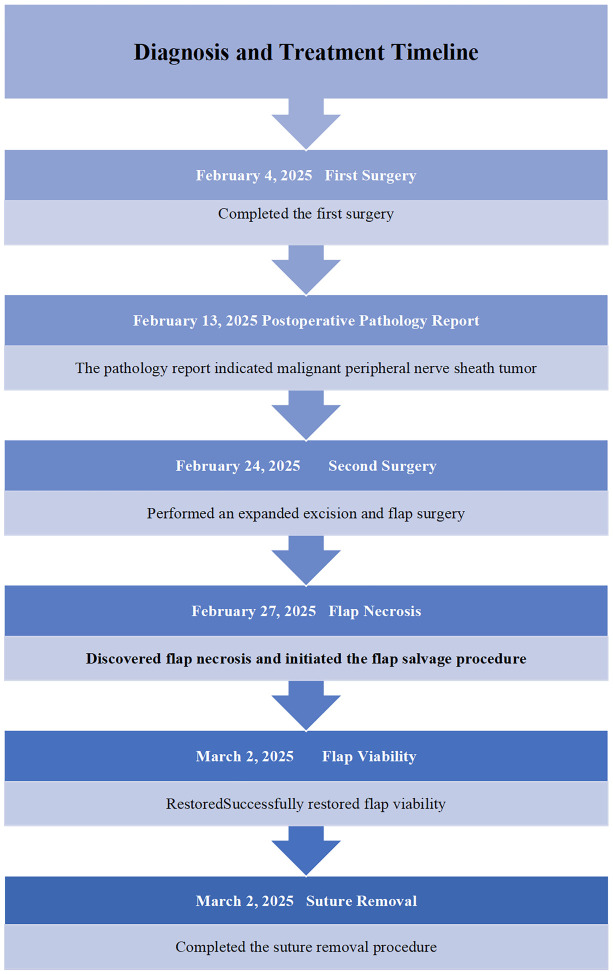

## 3 Discussion

### 3.1 MPNST diagnostic challenges and surgical management

The main clinical manifestations of MPNSTs include pain and numbness, however, these symptoms are non-specific, making it challenging to differentiate MPNSTs from other nerve-related lesions (5–8). Approximately 50% of MPNST cases are associated with neurofibromatosis type 1 (NF1), where neurofibromas, particularly plexiform neurofibromas (PNFs), have the potential to transform into MPNSTs. Additionally, around 10% of MPNST patients have a history of radiation exposure (2). PNFs and atypical neurofibromatous neoplasms of unknown biological potential (ANNUBPs) are significant high-risk factors for the development of MPNSTs. Patients with PNFs or ANNUBPs, especially those with NF1, should be closely monitored for signs of malignant transformation to ensure early detection and intervention (9). However, preoperative misdiagnosis remains common in atypical areas, such as the skin and subcutaneous tissue, where lesions may mimic benign entities like sebaceous cysts or lipomas, potentially delaying definitive management. This case underscores the critical need for pathological confirmation in patients with rapidly evolving masses in cases of clinical-imaging discordance.

For MPNST treatment, achieving R0 resection is crucial (10). While complete surgical resection is the primary treatment for MPNST it is often hindered by the large size of tumors, their proximity to complex nerve networks, and a low rate of negative resection margins. In this case, quadrant-based specimen orientation combined with intraoperative frozen section analysis enabled targeted excision until histologically negative margins were achieved. Preoperative ultrasound mapping of perforator vessels facilitated flap design while preserving vascular integrity. This integrated approach supports complex reconstruction while maintaining optimal oncologic safety.

### 3.1 Flap necrosis salvage: mechanistic rationale

Flap surgery is a crucial surgical technique for the repair of tissue defects resulting from tumor excision, trauma, vascular ulcers of the lower extremities, or skin loss due to diabetes, which plays a vital role in restoring both the structure and function of the affected area. However, it is associated with various complications, such as flap necrosis and wound dehiscence, with an incidence of up to around 50% (11). The primary mechanisms underlying flap necrosis are inadequate blood perfusion, impaired venous return, and ischemia-reperfusion injury (12). When the extent of ischemia exceeds the tissue’s tolerance threshold within a short period, and no intervention is made, the ischemic tissue will undergo irreversible necrosis.

Postoperative flap necrosis involving approximately 30% of the surface area on postoperative day (POD) 3 was managed using an integrative multimodal regimen combining Western pharmacotherapy and traditional Chinese medicine, alongside targeted local therapy. Papaverine is a non-opioid vasodilator that induces vasodilation by inhibiting cAMP/cGMP phosphodiesterase, increasing intracellular cyclic nucleotide levels to relieve microvascular spasm and enhance perfusion. Therefore, papaverine is especially important for flaps in ischemic conditions, as it can effectively improve tissue oxygenation and nutrient supply. In addition, papaverine alleviates ischemia-reperfusion injury by reducing the production of free radicals, decreasing intracellular calcium concentrations, and inhibiting inflammatory responses, further promoting flap survival and repair (13).

To augment its effects, we incorporated Danhong injection—a standardized traditional Chinese medicine formulation derived from *Salvia* and safflower—known for vasodilatory, anti-inflammatory, and pro-angiogenic properties in cardiovascular and cerebrovascular disorders (14–16). We think that the combination produces synergistic improvement in microcirculation. Externally, a biological enzyme gauze dressing was applied to maintain a moist wound-healing environment and promote tissue revitalization. Wet healing theory has been widely recognized in clinical practice and applied to wound treatment. Bioenzyme dressings have the ability to inhibit, stop bleeding, and promote wound healing due to their good biocompatibility, biodegradability, and biological functions. Conventional salvage strategies, including surgical revascularization, endovascular intervention, and systemic anticoagulation, primarily aim to restore macroscopic blood flow (17, 18). These methods are effective in restoring macroscopic blood flow but may be limited in cases with microvascular compromise, diffuse disease, or patients unsuitable for invasive interventions. Conventional techniques are considered the cornerstone of flap salvage. When inadequate perfusion is identified, correction of mechanical factors is generally prioritized, including suture release, hematoma evacuation, and surgical re-exploration when indicated. Adjunctive measures such as warming, administration of antispasmodic agents, and anticoagulant or antiplatelet therapy can be applied. These interventions are primarily directed at resolving vascular compromise and are regarded as first-line management.

This comprehensive treatment regimen achieved complete flap recovery by POD 9—substantially faster than typical recovery times reported with conventional therapy. We think that this regimen not only focuses on mechanical restoration of blood supply but also emphasizes microcirculatory regulation and wound bed optimization. To our knowledge, this is the first documented use of Danhong injection in postoperative flap salvage, representing an innovative application of combined Chinese and Western medical approaches to optimize microcirculatory restoration and reconstructive outcomes. Notably, the dosage and treatment duration should be adjusted on a case-by-case basis in accordance with patient-specific factors.

However, the follow-up period was limited to 3 months, which does not allow for assessment of long-term oncological safety, durability of flap survival, or recurrence risk. In addition, the comprehensive treatment regimen lacks standardized protocols, which may affect reproducibility and comparability. Although the treatment was well tolerated in this patient, potential drug interactions, adverse effects, and cost considerations warrant further evaluation. From the patient’s perspective, the treatment was reported as tolerable and acceptable, with subjective improvement in comfort and confidence during recovery; nevertheless, systematic incorporation of patient-reported outcomes will be important in future studies.

## 4 Conclusion

This case illustrates the diagnostic pitfalls of subcutaneous MPNSTs masquerading as infected cysts. The case highlights two key points (1): Clinicians are warned to expand the scope of differential diagnosis for subcutaneous masses that suddenly enlarge in elderly patients, especially when imaging is inconsistent with clinical manifestations, pathological examination should be performed as soon as possible to avoid misdiagnosis. If the mass is discovered during surgery, it is recommended to do frozen pathological examination in time (2). For evolving flap ischemia, we need timely multimodal intervention. The successful reversal of significant flap necrosis using triple therapy (papaverine, Danhong injection, and enzymatic debridement) offers a reference for wound management after similar plastic flap surgery. Further studies should explore the synergistic mechanisms of this pharmacologic approach in reconstructive surgery. This case contributes new insights and addresses an existing gap in the literature regarding similar instances.

## Data Availability

The raw data supporting the conclusions of this article will be made available by the authors, without undue reservation.
