# Supplementary material for: Salvage of necrotic flap following malignant peripheral nerve sheath tumor excision using multimodal pharmacotherapy: a case report
Source: Front Oncol. 2025 Oct 15;15:1689834. doi: 10.3389/fonc.2025.1689834 (PMC12568410; doi:10.3389/fonc.2025.1689834)
Supplement: Supplementary file 1 [file DataSheet1.pdf]

Supplementary Material

1. Supplementary Figures

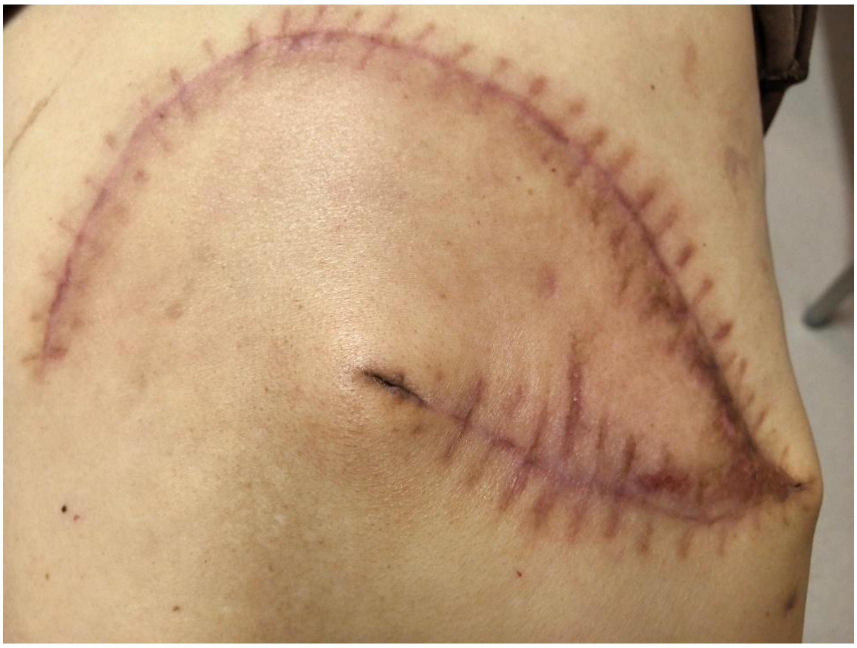

Supplementary Figure 1. Wound photograph at 3 months postoperatively.

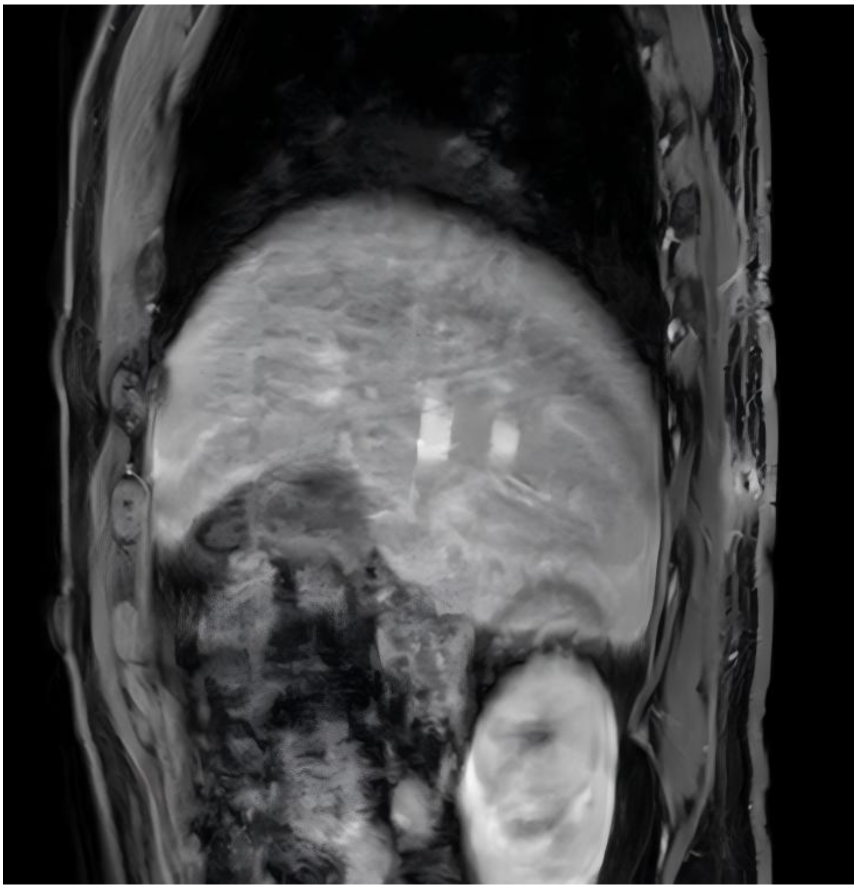

Supplementary Figure 2. MRI at 3 months postoperatively.
